# Preparation and Properties of CaCO_3_-Supported Nano-TiO_2_ Composite with Improved Photocatalytic Performance

**DOI:** 10.3390/ma12203369

**Published:** 2019-10-15

**Authors:** Jie Wang, Sijia Sun, Lei Pan, Zhuoqun Xu, Hao Ding, Wei Li

**Affiliations:** 1 Beijing Key Laboratory of Materials Utilization of Nonmetallic Minerals and Solid Wastes, National Laboratory of Mineral Materials, School of Materials Science and Technology, China University of Geosciences, Xueyuan Road, Haidian District, Beijing 100083, China; 3003170004@cugb.edu.cn (J.W.); 3003170003@cugb.edu.cn (S.S.); panleixx@outlook.com (L.P.); 2103170005@cugb.edu.cn (Z.X.); 2 Beijing Building Materials Academy of Sciences Research Co., Ltd., Shixing Street, Shijingshan District, Beijing 100041, China; ptliwei@163.com

**Keywords:** CaCO_3_–TiO_2_, composite particle, photocatalytic performance, recycling

## Abstract

In order to improve the photocatalytic degradation efficiency of nano-TiO_2_, reduce its usage and realize recycling and reuse, CaCO_3_–TiO_2_ composite photocatalyst was prepared with calcium carbonate (CaCO_3_) and TiO_2_ in a grinding machine through the integration of grinding depolymerization, dispersion and particle composition. The photocatalytic degradation performance, recycling performance, structure and morphology of CaCO_3_–TiO_2_ were studied. The interaction mechanism between CaCO_3_ and TiO_2_ and the improvement mechanism for the photocatalytic performance of TiO_2_ were also discussed. The results show that under the UV light irradiation for 20 and 40 min, the degradation efficiency of methyl orange by the composite photocatalyst with 40% TiO_2_ (mass fraction) was 90% and 100%, respectively. This was similar to that of pure TiO_2_, and the performance of the composite photocatalyst was almost unchanged after five cycles. CaCO_3_–TiO_2_ is formed by the uniform loading of nano-TiO_2_ particles on the CaCO_3_ surface, and the nano-TiO_2_ particles are well dispersed. Due to the facts that the dispersion of nano-TiO_2_ is improved in the presence of CaCO_3_ and the charge transport capability is improved through the interfacial chemical bonds between CaCO_3_ and TiO_2_, the formation of this complex is an intrinsic mechanism to improve the photocatalytic efficiency of nano-TiO_2_ and reduce its usage in application processes.

## 1. Introduction

In recent decades, with the rapid development of industries such as chemical industry, mining industry and agricultural breeding industry, environmental pollution problems, including water pollution, have become increasingly serious. Photocatalytic techniques are considered to be one of the most ideal ways to combat environmental pollution problems such as water pollution [1,2]. Therefore, it is of great significance to optimize the use conditions of photocatalysts, improving the function of photocatalyst technology and reducing the use amount of photocatalyst required to reduce the overall cost. Nano-TiO_2_ has become the most widely studied and applied semiconductor photocatalytic materials owing to its non-toxic, non-polluting and stable properties [3,4]. Under the excitation of ultraviolet light, electron–hole pairs are produced by electron transition in nano-TiO_2_, following which the separated electrons and holes migrate to the surface of TiO_2_ to produce active groups or directly react with pollutants. Thus, the degradation of pollutants such as dyes, heavy metal ions, phenols, dichlorophenol and antibiotics can be achieved through the further redox action [5,6,7,8]. However, there are still the several problems in the application of TiO_2_. Firstly, nano-TiO_2_ is easily agglomerated due to its high surface energy, which leads to the reduction of specific surface area and surface active sites, thus reducing its photocatalytic activity. Secondly, the loss of nano-TiO_2_ is serious when it is used in water, and is difficult to recover. The loss of nano-TiO_2_, caused by the lack of reuse, not only increases the use cost but also causes secondary pollution [9,10]. Many studies have shown that loading nano-TiO_2_ on the surface of the carrier with a large particle size to prepare a composite photocatalyst can effectively solve the above problems [11,12,13]. At present, the carriers for nano-TiO_2_ are mostly natural minerals and inorganic materials such as glass and ceramics. Among them, the natural mineral carriers that have been greatly studied are diatomite, zeolite, kaolin and montmorillonite [14,15,16,17]. However, these minerals generally need to be processed, such as via ore dressing and purification, to meet the requirements for catalyst carriers. This increases the cost of the composite photocatalyst, which undoubtedly restricts their industrial production and application.

Calcium carbonate (CaCO_3_) is an important inorganic mineral material, mainly made of non-metallic mineral raw materials such as calcite. It has the characteristics of high purity, high whiteness and low cost, and is obviously superior to most non-metallic minerals in cost-performance [18,19]. Therefore, the construction of CaCO_3_–nano-TiO_2_ composite photocatalyst using CaCO_3_ as a carrier can not only improve the recyclability and reusability performance of nano-TiO_2_, but also improve the photocatalytic efficiency of nano-TiO_2_ particles through loading to reduce the agglomeration of TiO_2_ particles. Meanwhile, the efficient utilization of CaCO_3_ mineral can be achieved by increasing its added value. Generally, TiO_2_-loaded mineral composite particles can be prepared by hydrolytic deposition, sol–gel and mechanical grinding methods. Sun et al. used purified diatomite as the carrier and prepared diatomite–nano-TiO_2_ composite photocatalyst by the hydrolysis of TiCl_4_ on its surface and the calcination of the product [20]. The maximum photocatalytic degradation efficiency of Rhodamine B (10 ppm) reached up to around 95% within 20 min of irradiation. Kun et al. prepared montmorillonite–nano-TiO_2_ composite photocatalyst by the sol–gel method with montmorillonite as the carrier [21]. The specific conversion of contaminant by the composite is three times that of TiO_2_. As for the loading of TiO_2_ on CaCO_3_, Tanabe [22] prepared CaCO_3_–TiO_2_ composite particles through carbonation in a TiO_2_ system. Sun [23] prepared CaCO_3_–TiO_2_ composite particles by the hydrophobic agglomeration method. However, these methods are complex and costly. Sun et al. prepared CaCO_3_–TiO_2_ composite pigment by liquid phase mechanical chemistry with CaCO_3_ as the carrier. However, this process requires a large amount of water, and subsequent processes such as filtration and drying are also required, which are complicated and consume more energy. Therefore, in this study, grinding depolymerization, dispersion and particle composition were integrated; the CaCO_3_–TiO_2_ composite photocatalyst was prepared by directly dry grinding CaCO_3_ and nano-TiO_2_ powder in a grinding machine, which has the advantages of requiring a simple process and low cost. In this study, the preparation conditions of the CaCO_3_–TiO_2_ composite photocatalyst were optimized. The morphology and structure of CaCO_3_–TiO_2_ composite particles were characterized and the photocatalytic degradation of CaCO_3_–TiO_2_ was carried out. The photocatalytic degradation of methyl orange by CaCO_3_–TiO_2_, including its reusability, was tested. Moreover, the combination mechanism between CaCO_3_ and TiO_2_ and the improvement mechanism for the photocatalytic performance of nano-TiO_2_ were discussed.

## 2. Methods

### 2.1. Raw Materials and Reagents

The CaCO_3_ raw material was calcite grinding powder produced in Jilin province, China, with a purity of 100% and whiteness of 95%. The D_97_ (particles with size ranging from 0 to D_97_ account for 97% of the weight ratio) of the used CaCO_3_ was 25 μm, 15 μm and 10 μm. The raw material of nano-TiO_2_ used in this study was the commercial degussa P_25_ product, which is composed of the mixed phase of anatase and rutile.

### 2.2. Preparation of CaCO_3_–TiO_2_ Composite Photocatalyst

According to the composite ratio, a certain amount of nano-TiO_2_ powder and CaCO_3_ powder with specific particle sizes were put into an oven and dried at 80 °C for 2 h. Then, the nano-TiO_2_ powder and CaCO_3_ powder were well mixed, and the mixed powder was added to an RK/XPM-Φ120 grinding machine. After grinding for 30 min (the diameter of grinding bowl was 120 mm, the speed was 9 r/min and the speed of the grinding rod was 220 r/min), the CaCO_3_–nano-TiO_2_ composite photocatalyst was obtained.

### 2.3. Photocatalytic Properties Test

The photocatalytic performance of CaCO_3_–TiO_2_ composite photocatalyst was tested with methyl orange as the target degradation product. A 300 W mercury lamp with a dominant wavelength of 254 nm was used as the light source. First, 50 mg of CaCO_3_–TiO_2_ composite photocatalyst was added to 50 mL of as-prepared methyl orange solution (the concentration was marked as C_0_, 10 mg/L) to obtain a suspension. In order to reduce the measurement error caused by sample adsorption, dark reaction was carried out for 1 h. After turning on the light source, the residual concentration of methyl orange was tested at specified intervals and marked as C. The photocatalytic degradation performance of the samples was characterized and evaluated by the change of C/C_0_. Linear regression was performed on the relationship between –Ln (C/C_0_) and t (lighting time), and the linear slope was used to characterize the photocatalytic degradation rate.

The measurement of the concentration of methyl orange solution after photodegradation was as follows: the solution was centrifugated, the supernatant was removed and its absorbance was measured by a UV-VIS spectrophotometer (Cary 5000, Varian company, Palo Alto, CA, USA). The concentration of methyl orange in the solution was obtained through the relationship between the absorbance and concentration.

After the photocatalytic degradation of methyl orange, the suspension was centrifuged to obtain the sediments (photocatalyst). After being dried at a low temperature, the composite photocatalyst was tested for the photocatalytic degradation performance again, which was regarded as one cycle. The above process was repeated multiple times to obtain the cycle degradation performance of the samples.

### 2.4. Characterization

An X-ray powder diffractometer (D/MAX2000, Rigaku Corporation, Tokyo, Japan) was used to analyze the phase composition of the CaCO_3_–TiO_2_ composite photocatalyst. A scanning electron microscope (SEM, S-3500N, HITACHI, Tokyo, Japan) was used to observe the sample morphology. The UV-VIS diffuse reflection absorption spectrum (Cary 5000, USA Varian, Palo Alto City, CA, USA) was used to characterize the light absorption properties of the samples. Fourier transform infrared spectroscopy (Spectrum 100, PerkinElmer Instruments (Shanghai) Co., Ltd., Shanghai, China) was used to test the binding properties of CaCO_3_ and TiO_2_.

## 3. Results and Discussion

### 3.1. Photocatalytic Degradation of Methyl Orange by CaCO_3_–TiO_2_

#### 3.1.1. Effect of Particle Size of CaCO_3_

**Figure 1:**
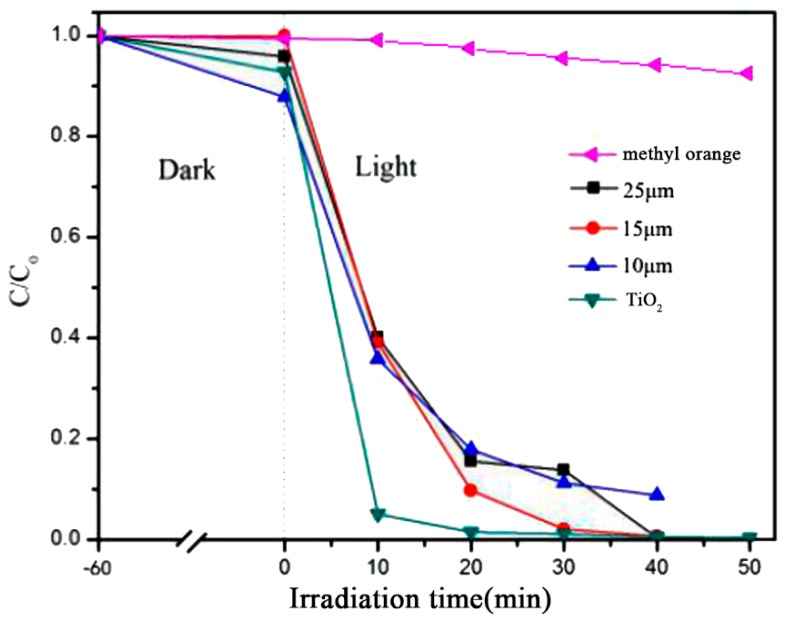
Effect of the particle size of CaCO_3_ on the performance of the composite photocatalyst.

In order to investigate the effect of the particle size of CaCO_3_ on the properties of the prepared CaCO_3_–TiO_2_ composite photocatalyst, CaCO_3_–TiO_2_ composite photocatalysts were prepared with 25 μm, 15 μm and 10 μm CaCO_3_ as the carrier (the mass ratio of nano-TiO_2_ was 40%). Figure 1 shows the photocatalytic degradation of methyl orange by CaCO_3_–TiO_2_ and pure TiO_2_, and a blank experiment was also conducted. It can be seen that when the pure methyl orange solution was illuminated by the ultraviolet light only, the C/C_0_ remained almost unchanged with the increase of illumination time, indicating that there was no degradation effect on the methyl orange. By contrast, treatment with the CaCO_3_–TiO_2_ composite photocatalyst (the size of CaCO_3_ was 15 μm) and pure TiO_2_ both resulted in the effective degradation of methyl orange. After 30 min of irradiation, the C/C_0_ was reduced to less than 0.05, indicating that the degradation efficiency was higher than 95%; after 40 min of irradiation, the degradation efficiency reached 100%. Obviously, CaCO_3_–TiO_2_ has excellent photocatalytic performance, which is similar to that of nano-TiO_2_. Since the mass ratio of TiO_2_ to CaCO_3_–TiO_2_ was only 40%, the above results indicate that CaCO_3_ has an improving effect on the performance of TiO_2_.

Figure 1 also shows that among the three CaCO_3_–TiO_2_ samples, the composite with 15 μm CaCO_3_ as the carrier exhibited the highest degradation efficiency. After 20 min of irradiation, the degradation efficiency of methyl orange was 90%, and the samples with 25 μm and 10 μm CaCO_3_ as the carrier only reached about 82%. When the irradiation time increased to 30 min, the methyl orange degradation efficiencies of the three samples were 98%, 86% and 89%, respectively. The above results indicate that the particle size and specific surface area of CaCO_3_ are important, and that CaCO_3_ with a particle size of 15 μm properly matched with the nanoTiO_2_ particles to form an optimal composite relationship. Therefore, CaCO_3_ with a particle size of 15 μm was chosen as the carrier to be compounded with nano-TiO_2_.

#### 3.1.2. Effect of Loading Amount of Nano-TiO_2_

**Figure 2:**
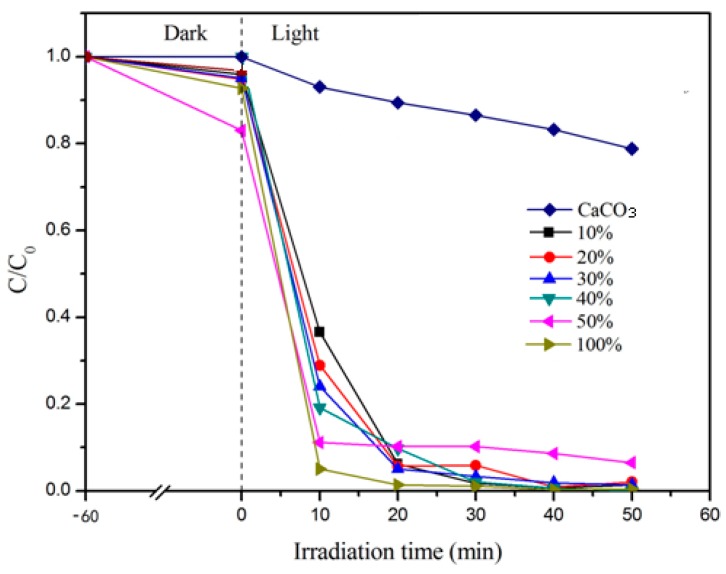
Effect of mass ratio of TiO_2_ on the properties of the composite photocatalyst.

As the photocatalytic active component in CaCO_3_–TiO_2_, the loading amount of nano-TiO_2_ should have a significant effect on the performance of the composite photocatalyst. Therefore, using 15 μm CaCO_3_ as the carrier, CaCO_3_–TiO_2_ composite photocatalysts with different mass ratios of nano-TiO_2_ were prepared, and the degradation performance of methyl orange under ultraviolet light was tested. The results are shown in Figure 2. As the mass ratio of nano-TiO_2_ increased from 10% to 40%, the degradation efficiency of CaCO_3_–TiO_2_ increased gradually. When the composite photocatalyst with a TiO_2_ mass ratio of 40% was illuminated for 20 min, the C/C_0_ was lower than 0.1, indicating that the degradation efficiency reached higher than 90%. And when the irradiation time increased to 40 min, the degradation efficiency reached 100%, which is equivalent to pure TiO_2_. When the mass ratio of nano-TiO_2_ increased to 50%, although the degradation efficiency was improved after 10 min of illumination, the degradation efficiency remained almost unchanged when the irradiation time was extended, with a maximum of only 90%. Obviously, the mass ratio of nano-TiO_2_ in CaCO_3_–TiO_2_ should be set as 40%.

Since CaCO_3_–TiO_2_ with only a small mass ratio of nano-TiO_2_ reaches the photocatalytic degradation effect similar to that of pure nano-TiO_2_, the use amount of TiO_2_ can be significantly reduced and thus cost can be saved in practical applications. This is a result of the significant improvement in the photocatalytic efficiency of TiO_2_ by its combination with CaCO_3_. It is speculated that this improvement results from the improvement of the dispersibility of nano-TiO_2_ and the formation of a combination interface between TiO_2_ and CaCO_3_.

#### 3.1.3. Photocatalytic Degradation of Different Concentrations of Methyl Orange Solution by CaCO_3_–TiO_2_

**Figure 3:**
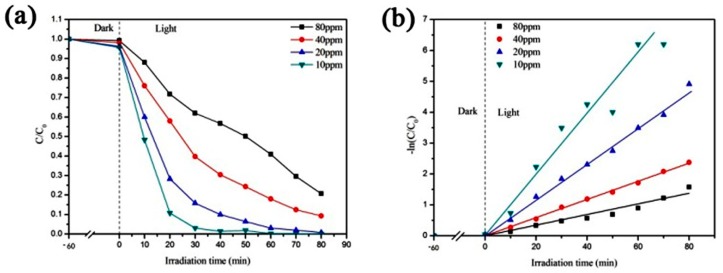
Effect of the concentration of methyl orange solution on the degradation efficiency (**a**) and degradation rate (**b**) of CaCO_3_–TiO_2_ composite photocatalyst.

The investigation of the degradation performance of CaCO_3_–TiO_2_ in different concentrations of methyl orange solution can not only further verify the photocatalytic degradation ability of CaCO_3_–TiO_2_, but it can also determine its reasonable scope of application. Figure 3 shows the photocatalytic degradation effect of CaCO_3_–TiO_2_ for different concentrations of methyl orange solution. It can be seen that the degradation efficiency of the CaCO_3_–TiO_2_ composite photocatalyst on each concentration of methyl orange solution increased with the increase of the illumination time, and finally reached a higher degradation effect. Comparing the degradation effect of different concentrations, it was found that the degradation efficiency of methyl orange solution with concentrations of 10 and 20 ppm by CaCO_3_–TiO_2_ was higher than that of concentrations of 50 and 80 ppm. Among them, the methyl orange solution with concentrations of 10 and 20 ppm could be completely degraded (C/C_0_ = 0) by CaCO_3_–TiO_2_ at illumination times of 60 and 80 min, respectively, and the degradation rate was faster. In contrast, the degradation rates of 50 and 80 ppm methyl orange solution by CaCO_3_–TiO_2_ were reduced, and the degradation efficiencies were 90% and 80%, respectively (C/C_0_ was 0.1 and 0.2, respectively) at illumination times of 60 and 90 min. These results show that the CaCO_3_–TiO_2_ composite photocatalyst can effectively degrade methyl orange solution with concentrations of 10–80 ppm. The reason why the degradation rate decreases with the increasing concentration of methyl orange solution is that high concentrations of pollutants require longer reaction times.

#### 3.1.4. Recycling Performance of CaCO_3_–TiO_2_ Composite Photocatalyst

**Figure 4:**
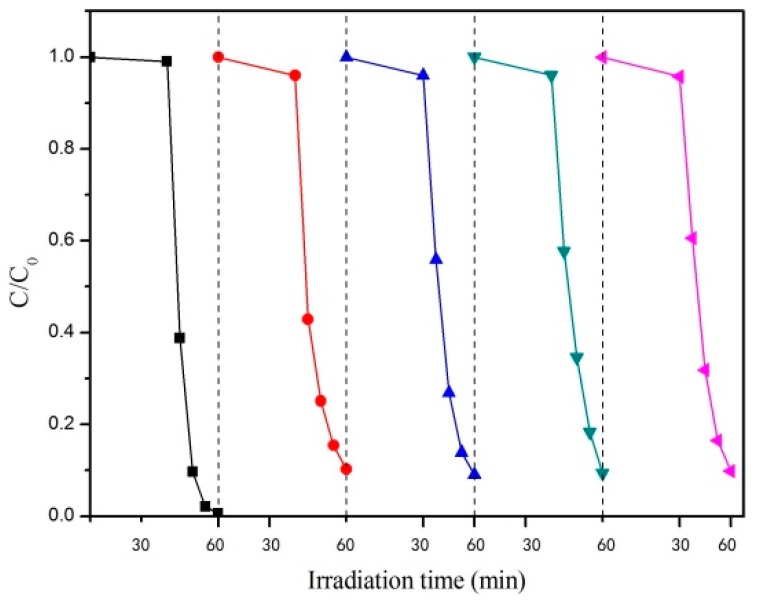
Cyclic curve of the degradation of methyl orange by CaCO_3_–TiO_2_ composite photocatalyst.

The recycling performance of the catalyst is an important factor reflecting its stability and practical application performance. Therefore, we investigated the recycling performance of the optimum CaCO_3_–TiO_2_ composite photocatalyst (the particle size of CaCO_3_ was 15 μm, the mass ratio of TiO_2_ was 40%). The results are shown in Figure 4. The results show that the photocatalytic degradation performance of CaCO_3_–TiO_2_ after five cycles is similar to that of the first use, indicating that recycling does not reduce its performance. This indicates that the CaCO_3_–TiO_2_ composite photocatalyst prepared in this study has excellent recycling performance and can be recycled and reused several times, thereby reducing the cost. Obviously, this is due to the loading of TiO_2_ on the surface of CaCO_3_, which results in the nano-TiO_2_ being firmly fixed on the surface of CaCO_3_. Therefore, the loss of TiO_2_ is prevented, and CaCO_3_–TiO_2_ can easily be separated from water due to its large particle size. However, single nano-TiO_2_ particles have a small particle size, so they are difficult to precipitate and easily lost when separated from their carrier. Therefore, it is believed that the CaCO_3_–TiO_2_ composite photocatalyst has practical application prospects for sewage treatment.

### 3.2. Optical Performance Test

**Figure 5:**
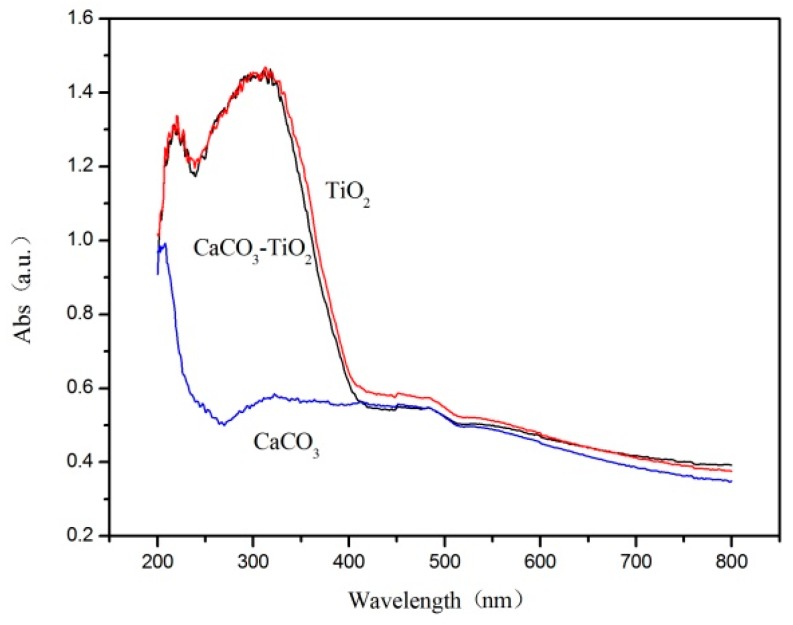
UV-VIS absorption spectra of CaCO_3_, TiO_2_ and CaCO_3_–TiO_2_.

Figure 5 shows the UV-VIS diffuse reflectance absorption spectra (DRS) of the CaCO_3_–TiO_2_ composite photocatalyst as well as the raw materials of CaCO_3_ and TiO_2_. It can be seen that CaCO_3_–TiO_2_ and TiO_2_ exhibit similar light absorption characteristics. They show no light absorption in the visible light region (wavelengths of 400–800 nm) and show strong absorption in ultraviolet light (wavelengths of 200–400 nm), which is consistent with the bandgap characteristics of the TiO_2_ semiconductor (3.2 eV of bandgap width). By contrast, CaCO_3_ shows almost no absorption of both visible light and ultraviolet light. The results show that CaCO_3_–TiO_2_ has similar properties to TiO_2_, consistent with the observed strong photocatalytic degradation performance of CaCO_3_–TiO_2_ and TiO_2_. Meanwhile, it is inferred that a composite structure of CaCO_3_ with surface-loaded nano-TiO_2_ is formed in the creation of CaCO_3_–TiO_2_.

### 3.3. Morphology and Structure of CaCO_3_–TiO_2_ Composite Photocatalyst

#### 3.3.1. SEM Analysis

**Figure 6:**
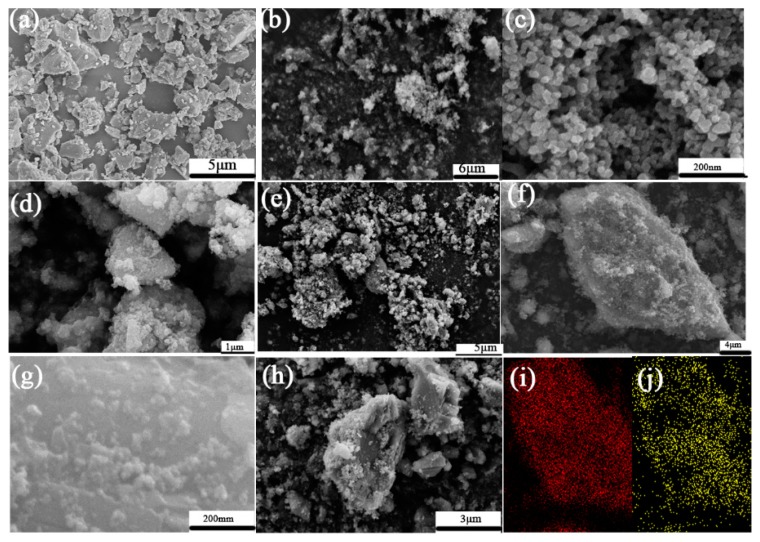
SEM images of CaCO_3_ (**a**), TiO_2_ (**b**,**c**), CaCO_3_–TiO_2_ composite photocatalyst with TiO_2_ mass ratios of 20% (**d**), 40% (**e**), 50% (**f**,**g**). The mixture of CaCO_3_ and TiO_2_ (**h**). The mapping of Ca (**i**) and Ti (**j**) corresponding to (**f**).

Figure 6 shows SEM images of CaCO_3_, nano-TiO_2_ raw materials, CaCO_3_–TiO_2_ composite photocatalyst with different mass ratios of TiO_2_ and the physical mixtures of CaCO_3_ and TiO_2_ (40% TiO_2_ mass ratio). Figure 6a shows that the CaCO_3_ particles have a bulk shape with a particle size of about 1–3 μm, as well as smooth surfaces without coatings. Figure 6b,c shows that the nano-TiO_2_ particles are fine, but their agglomeration is severe, with an aggregate size of up to 2 μm. Figure 6d–g show that when CaCO_3_ is combined with nano-TiO_2_, the surface of CaCO_3_ particles is uniformly covered by fine particles, and the surface becomes rough. Figure 6i,j are the mapping results of Ca and Ti elements corresponding to Figure 6f; it can be seen that Ca distributes in the particle contour range of CaCO_3_, reflecting the characteristic of CaCO_3_. The distribution of Ti in the scanning area is uniform, which corresponds to the location of the particles. This proves that the TiO_2_ is distributed on the surface of CaCO_3_ uniformly. Obviously, the CaCO_3_–TiO_2_ composite photocatalyst is composed of composite particles characterized by nano-TiO_2_ loaded on the CaCO_3_ surface. Therefore, it is inferred that CaCO_3_–TiO_2_ should exhibit the properties of nano-TiO_2_ (photocatalytic properties). In addition, it was found that the dispersion of TiO_2_ loaded on the surface of CaCO_3_ was significantly higher than that of TiO_2_ alone. The particle unit size is generally reduced to less than 0.2 μm, which allows the active sites of TiO_2_ to be more exposed, thereby increasing its photocatalytic performance. This is undoubtedly the intrinsic mechanism enabling CaCO_3_–TiO_2_ to exhibit a photocatalytic degradation performance comparable to that of pure TiO_2_ when the mass ratio of TiO_2_ is only 40%.

Figure 6d–f shows that as the mass ratio of TiO_2_ increased from 20% to 50%, the surface of the CaCO_3_ was coated by nano-TiO_2_ particles more and more uniformly and completely. Especially when the mass ratio of TiO_2_ was 40%, the surface of CaCO_3_ was almost completely covered by TiO_2_ particles. However, in the physical mixture of CaCO_3_ and TiO_2_ with the same composite proportion (Figure 6h), the loading of nano-TiO_2_ was very poor. Only a small part of the surface of CaCO_3_ particles contained loadings, and most of them were bare. The above results indicate that the co-grinding of CaCO_3_ and TiO_2_ greatly promotes the loading function, thereby improving the performance of CaCO_3_–TiO_2_.

#### 3.3.2. XRD Analysis

**Figure 7:**
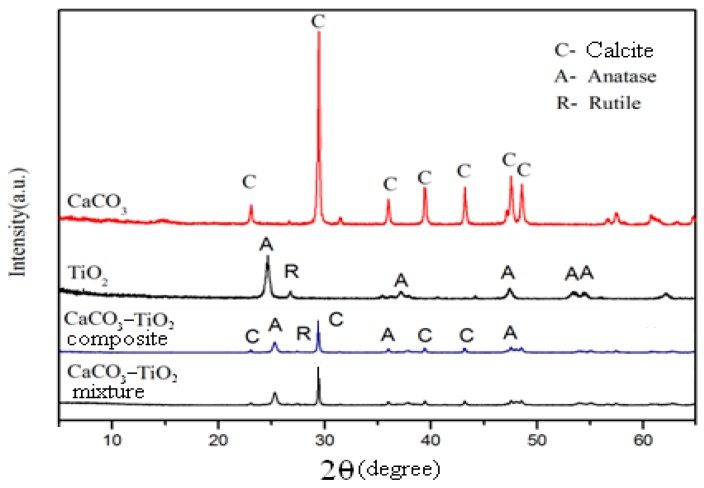
X-ray diffraction (XRD) spectra of CaCO_3_, TiO_2_, CaCO_3_–TiO_2_ composite and the physical mixture of CaCO_3_ and TiO_2_.

Figure 7 displays the XRD spectra of CaCO_3_, nano-TiO_2_ raw material, CaCO_3_–TiO_2_ composite photocatalyst and the physical mixture of CaCO_3_ and TiO_2_. The results reveal that only the characteristic diffraction peaks of calcite appeared in the XRD spectrum of CaCO_3_, indicating its high purity. The characteristic diffraction peaks of rutile and anatase appeared in the spectrum of nano-TiO_2_, indicating that it consisted of rutile and anatase mixed phase, consistent with the phase composition of P_25_. In the spectrum of the CaCO_3_–TiO_2_ composite photocatalyst, only calcite, rutile and anatase crystal phases were observed, indicating that the compounding of CaCO_3_ and TiO_2_ does not produce a new phase. It was concluded that the combination of CaCO_3_ and TiO_2_ occurs in their interface region, and the binding properties should be chemical or physical action within the interface area. The phase composition of the physical mixture of CaCO_3_ and TiO_2_ was found to be the same as that of CaCO_3_–TiO_2_ composite photocatalyst, but the intensity of the characteristic peaks of CaCO_3_ in CaCO_3_–TiO_2_ composite photocatalyst was weaker than that in the physical mixture. This may be due to the reduction of CaCO_3_ exposure in CaCO_3_–TiO_2_ due to the coating of the surface with titanium dioxide, while most of the surface of CaCO_3_ in the physical mixture was still bare [23]. The above results also indicate the successful compounding of CaCO_3_ and TiO_2_ in CaCO_3_–TiO_2_.

#### 3.3.3. Infrared Spectrum Analysis

**Figure 8:**
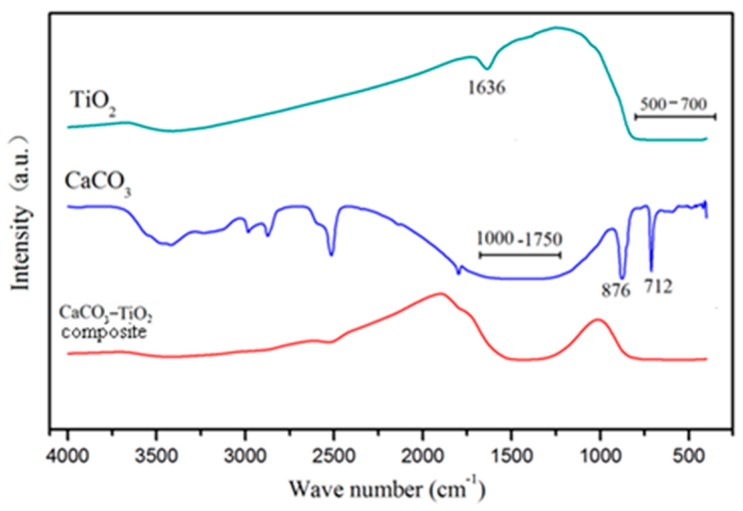
FT-IR spectra of CaCO_3_, TiO_2_ and CaCO_3_–TiO_2_ composite.

The infrared spectra of CaCO_3_, nano-TiO_2_ raw material and CaCO_3_–TiO_2_ composite photocatalyst were measured, as shown in Figure 8. In the infrared spectrum of nano-TiO_2_, the wide absorption band in the range of 3250–3700 cm^−1^ and the absorption peak at 1636 cm^−1^ respectively represent the stretching vibration and bending vibration of the O–H bond in the water molecules and hydroxyl groups on the surface of TiO_2_. The absorption band in the range of about 3300–3500 cm^−1^ in the CaCO_3_ spectrum represents the characteristics of adsorption water and hydroxyl groups on its surface. In the infrared spectrum of the CaCO_3_–TiO_2_ composite photocatalyst, the absorption peak at 1636 cm^−1^ of TiO_2_ disappeared, and the absorption peak of CaCO_3_ ranging from 3300 to 3500 cm^−1^ was weakened. It has been suggested that CaCO_3_ and TiO_2_ form a firm chemical combination through their surface hydroxyl groups [24].

**Figure 9:**
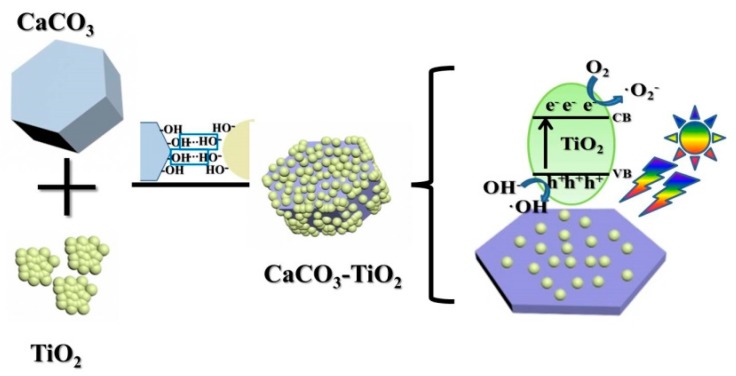
Improvement mechanism of photocatalytic performance.

Based on the above study, the mechanism of CaCO_3_ as a carrier to improve the photocatalytic performance and utilization efficiency of nano-TiO_2_ can be summarized as follows: (1) The dispersibility of TiO_2_ is improved by uniformly loading TiO_2_ on the surface of CaCO_3_, thereby causing an increase in active sites and light absorption areas. (2) A chemical bond is formed between TiO_2_ and CaCO_3_ at their interface, which provides a new transport channel for the photo electrons [25,26], and further improves the separation efficiency of electrons and holes. (3) In CaCO_3_–TiO_2_, nano-TiO_2_ and CaCO_3_ are firmly combined due to their surface chemical bonding, which prevents the loss of TiO_2_ when degrading pollutants in water [9,27]. Therefore, this composite photocatalyst can be easily recovered and recycled from water by sedimentation, thereby reducing the use amount of TiO_2_ as well as the cost. Figure 9 is a schematic diagram reflecting the above principles.

## 4. Conclusions

(1) The CaCO_3_–TiO_2_ composite photocatalyst was prepared by the co-grinding of CaCO_3_ and TiO_2_. The optimal CaCO_3_–TiO_2_ composite photocatalyst, in which the particles size of CaCO_3_ is 15 μm and the mass ratio of TiO_2_ is 40%, shows excellent photocatalytic degradation performance towards methyl orange and good recovery performance. The degradation efficiency of optimal CaCO_3_–TiO_2_ composite photocatalyst was found to be 90% and 100% after 20 and 40 min of ultraviolet light illumination, respectively. The degradation effect is comparable to pure TiO_2_. Moreover, its degradation effect on methyl orange is not significantly reduced after five cycles.
(2) CaCO_3_–TiO_2_ composite photocatalyst is characterized by CaCO_3_ loaded by nano-TiO_2_ uniformly and completely. The dispersibility of the loaded TiO_2_ is significantly enhanced compared to pure TiO_2_, and a strong chemical bond is formed between CaCO_3_ and TiO_2_ particle interfaces. These are important mechanisms for improving the photocatalytic efficiency of nano-TiO_2_ and reducing the its amount in CaCO_3_–TiO_2_.
